# A survey on how preregistration affects the research workflow: better science but more work

**DOI:** 10.1098/rsos.211997

**Published:** 2022-07-06

**Authors:** Alexandra Sarafoglou, Marton Kovacs, Bence Bakos, Eric-Jan Wagenmakers, Balazs Aczel

**Affiliations:** ^1^ Department of Psychology, University of Amsterdam, Amsterdam, The Netherlands; ^2^ Doctoral School of Psychology, ELTE Eotvos Lorand University, Budapest, Hungary; ^3^ Institute of Psychology, ELTE Eotvos Lorand University, Budapest, Hungary

**Keywords:** open science, meta-science, replication crisis

## Abstract

The preregistration of research protocols and analysis plans is a main reform innovation to counteract confirmation bias in the social and behavioural sciences. While theoretical reasons to preregister are frequently discussed in the literature, the individually experienced advantages and disadvantages of this method remain largely unexplored. The goal of this exploratory study was to identify the perceived benefits and challenges of preregistration from the researcher’s perspective. To this end, we surveyed 355 researchers, 299 of whom had used preregistration in their own work. The researchers indicated the experienced or expected effects of preregistration on their workflow. The results show that experiences and expectations are mostly positive. Researchers in our sample believe that implementing preregistration improves or is likely to improve the quality of their projects. Criticism of preregistration is primarily related to the increase in work-related stress and the overall duration of the project. While the benefits outweighed the challenges for the majority of researchers with preregistration experience, this was not the case for the majority of researchers without preregistration experience. The experienced advantages and disadvantages identified in our survey could inform future efforts to improve preregistration and thus help the methodology gain greater acceptance in the scientific community.

A physicist had a horseshoe hanging on the door of his laboratory. His colleagues were surprised and asked whether he believed that it would bring luck to his experiments. He answered: ‘No, I don’t believe in superstitions. But I have been told that it works even if you don’t believe in it.’

[1, p. 14]

Over the past decade, the social sciences have undergone a methodological metamorphosis. In order to increase the quality and credibility of confirmatory empirical research, both journals and researchers have adopted a series of methodological reform measures [2,3]. Among these reform measures, preregistration is arguably the most consequential. The preregistration of empirical studies entails the specification of the research design, the hypotheses and the analysis plan before data is collected and analysed. Preregistration protects the confirmatory status of the reported results by preventing biases—such as confirmation bias and hindsight bias—from contaminating the statistical analysis [4,5].

The concept of preregistration is not new; as early as 1878, Peirce [6, p. 476] established three rules to guarantee that a hypothesis leads to a probable result, the first being that a hypothesis should be explicitly stated before data are collected to test its truth. In some research areas, such as medical clinical trials, preregistration has long become scientific routine. For instance, in the world’s highest impact journal, the *New England Journal of Medicine*, the registration of clinical trials is a prerequisite for publication. A recent interdisciplinary study by Malički *et al.* [7] shows that while preregistration receives the least support by researchers in a catalogue of responsible research practices, as many as 39% of researchers within the health sciences agreed with the statement that all studies should be preregistered (compared to 17% of researchers in other fields).^1^

In the last 10 years, preregistration has also found its way into psychological science. In fact, preregistration has become so widespread that some believe it is on its way to becoming the norm [8]. The number of preregistrations has increased at ‘unprecedented and accelerating rates’ [8, p. 19]. For instance, a recent survey among researchers in the Netherlands found that 38.9% of researchers in the social and behavioural sciences had preregistered a study before [9]. Online repositories have been created to store preregistrations (e.g. the Open Science Framework (OSF; https://osf.io) and AsPredicted.org), and several journals recognize preregistered studies with badges [10]. In addition, over 300 journals now offer the Registered Reports format as a submission option, allowing authors to integrate preregistration with the peer-review process ([11,12]; https://osf.io/rr/).

In the course of its rapid spread, however, the effectiveness of preregistration has been repeatedly questioned. When discussing ways to combat the crisis of confidence, critics have argued that too heavy an emphasis is being placed on methodological reforms (e.g. [13–16]). Preregistration was not designed to improve the theoretical foundation of studies. Instead, it was proposed to limit the degrees of freedom researchers have in designing and executing studies, and analysing the results. For that reason, critics argue that strong theory development, more so than methodological reforms, would advance psychological science in the long term. That is, if predictions were derived from weak theories, even the application of the most rigorous methods will not produce reliable scientific results. For instance, if theories do not adequately define the conditions under which a particular phenomenon is observed, it remains unclear whether a non-significant result constitutes evidence against the theory or whether the chosen operationalizations were inappropriate [14]. Thus, instead of focusing primarily on the prevention of questionable research practices, the discussion on how to improve psychological science should be dominated by topics such as theory development, good experimental designs and the proper statistical modelling of theoretical predictions [14–17].

In defense of preregistration, van’t Veer & Giner-Sorolla [18] argued that while preregistration might not *directly* improve theory development, preregistration will help shift the research focus away from the evaluation of a consistent and statistically significant pattern of results and towards the assessment of theory and methods. In addition, van’t Veer & Giner-Sorolla [18] argue that preregistration may lead to positive side-effects that improve the overall quality of the scientific product. For instance, since all team members need to approve and scrutinize the hypotheses, methods, and analyses before data collection, study preregistration would improve the collaboration within the team and therefore yield more carefully thought-out research plans. However, it is still unclear whether or to what extent researchers actually perceive preregistered studies to be of higher quality than non-preregistered studies. On the one hand, Alister *et al.* [19] found that researchers reported that they would be more confident that a finding would replicate when the original authors had adhered to open science practices such as preregistration. On the other hand, a study by Field *et al.* [20] found only ambiguous evidence that researchers trust in preregistered empirical findings more than non-preregistered ones.

It has been argued that the scrutiny associated with preregistration might even harm certain aspects of the research workflow. For instance, preregistration can be effortful and time-consuming (e.g. [8,18]). Open research practices were also found to have a small but statistically significant association with work pressure [9]. As recognized by Nosek *et al.* [21] ‘[p]reregistration requires research planning and it is hard, especially contingency planning. It takes practice to make design and analysis decisions in the abstract, and it takes experience to learn what contingencies are most important to anticipate. This might lead researchers to shy away from preregistration for worries about imperfection’ (p. 817). Note that other researchers have claimed the exact opposite, namely that preregistration is easy [22] and that the Registered Report format saves time [20].

To date there does not exist an empirical assessment about the experiences and expectations that researchers have concerning the impact of preregistration on their workflow. This study seeks to chart the perceived benefits and drawbacks of preregistration so we may learn what motivates researchers to adopt this practice and possibly also what prevents researchers from adopting it. At the same time, researchers’ past experiences with preregistration may be informative for pragmatic would-be adopters. This study concerns two groups of researchers: those who published both preregistered studies and non-preregistered studies and those who only published non-preregistered studies.

## 1. Disclosures

### 1.1. Data, materials and preregistration

The current study was preregistered on the Open Science Framework; in our project folder, readers can access the preregistration, as well as all materials for both the pilot and the main survey, the contact database used for the main survey, the anonymized raw and processed data (including relevant documentation), and the R code to conduct all analyses (including all figures; see table 1 for an overview of URLs for the different resources). In our data, identifying information such as names and affiliations of the respondents were removed. Any deviations from the preregistration are mentioned in this manuscript. Note that we removed email addresses from the contact database for privacy reasons.

**Table 1.** Overview of URLs to this study’s materials available on the Open Science Framework.

resource	URL
project page	https://osf.io/jcdvb/
preregistration of main study	https://osf.io/qezv5/
preregistration of pilot study	https://osf.io/g3fv7/
data and analysis code	https://osf.io/5ytpk/
surveys	https://osf.io/dzybn/
ethics documents	https://osf.io/atgb7/

### 1.2. Reporting

We report how we determined our sample size, all data exclusions, all manipulations, and all measures in the study.

### 1.3. Ethical approval and participant compensation

The study was approved by the local ethics board of the University of Amsterdam (registration no. 2019-PML-11423) and of the Eotvos Lorand University (registration no. 2019/17). All participants were treated in accordance with the Declaration of Helsinki. Researchers who participated in the survey were given the opportunity to enter a raffle for a voucher from a webshop of their choice.

## 2. Methods

### 2.1. Pilot study and creating materials

Before conducting the main survey, we conducted a pilot study to determine the aspects of the research workflow that are most affected by preregistration. For this pilot study, we contacted 176 researchers from our database (described in the following sections) and asked them how their preregistered studies differed from their non-preregistered studies in terms of workflow, data management and scientific quality. Respondents were asked to list both advantages and disadvantages in a free-text format. In total, we received answers from 49 researchers. The answers were then categorized by three of the authors (A.S., B.A. and M.K.). In total, nine aspects of the research process were identified as being especially impacted by preregistration. These aspects of the research process were then included as items in the main survey.

### 2.2. Participants

The researchers in the preregistration group were recruited based on a contact database of published preregistered studies. Initially, we created a collection of 711 research articles in which the authors referred to a preregistered analysis plan. This collection of studies consisted of 404 preregistered and published articles that were part of the bibliographical collection of published preregistered articles from the Center of Open Science, 128 articles mentioned in Akker *et al.* [23] which originated from a database of articles with open science badges by Kambouris *et al.* [24], 22 articles based on a collection from Schäfer & Schwarz [25] and 157 articles based on a non-systematic collection of the present authors. From this initial collection of articles, we then excluded non-empirical studies (e.g. meta-analyses), Registered Reports, articles that did not include a URL to their preregistration, articles whose preregistration has been published on platforms other than the OSF (e.g. AsPredicted.org), and duplicates. This left a final sample of 487 articles from which we extracted the email addresses of the corresponding authors.

### 2.3. Sampling plan

No sample size target was specified for the preregistration group; we contacted all authors from our contact database. For the non-preregistration group, we preregistered that data would be collected until we reached a sample size as large as at least 90% of the sample size from the preregistration group. As will be discussed in the section ‘Sample characteristics’, we were unable to reach that goal.

### 2.4. Materials

The survey was generated using the online survey software Qualtrics [26]. The items in the main survey were based on the results of the pilot study and a discussion among the authors. The survey included questions about (1) the nine aspects of the research process that were identified in the pilot study; (2) the respondents’ general opinion about preregistration and (3) the respondents’ research background. Respondents from the preregistration group were instructed to relate the questions to their own *experience* (i.e. Please indicate below how you believe preregistration has affected your work.), whereas researchers from the non-preregistration group were instructed to indicate their *expectations* about preregistration (e.g. Please indicate below how you believe preregistration would affect your work.). Finally, respondents also had the opportunity to give feedback on the survey and provide us with free-text on the topic of preregistration.

#### 2.4.1. Nine aspects of research process

Respondents were asked to indicate whether preregistration has benefited or harmed (preregistration group) or would benefit or harm (non-preregistration group) the nine aspects of the research process listed in table 2. For each question, respondents could also select the options *I do not know* and *Not applicable*.

**Table 2.** Nine aspects of the research process included in the survey as presented to the preregistration group. Respondents were asked to indicate on the following 1 to 7 scales, how they believed preregistration has affected their work. Researchers in the non-preregistration group were asked how they believed preregistration *would* affect each aspect.

	response anchors of the 7-point rating scales	
due to preregistration, the	(1)	(7)
analysis plan	*got less thought-through*	*got more thought-through*
research hypothesis	*got less thought-through*	*got more thought-through*
experimental design	*got less thought-through*	*got more thought-through*
preparatory work (e.g. pilot or simulation studies)	*got worse*	*improved*
data management	*got less thought-through*	*got more thought-through*
project workflow	*got less thought-through*	*got more thought-through*
collaboration in the team	*got worse*	*got better*
work-related stress	*was increased*	*was reduced*
total project duration	*was longer*	*was shorter*

#### 2.4.2. Opinion about preregistration

Three items asked respondents about their general opinion concerning preregistration. The first item asked about whether respondents thought preregistration has made it easier (preregistration group) or would make it easier (non-preregistration group) to avoid questionable research practices. The item was answered using a 7-point Likert scale from 1 (*Very Strongly Disagree*) to 7 (*Very Strongly Agree*). The second item asked how often respondents would consider preregistration in their future work. The item was answered using a 7-point Likert scale from 1 (*Always*) to 7 (*Never*). The third item asked about whether respondents would recommend preregistration to other researchers in their field. The item was answered using a 7-point Likert scale from 1 (*Very Strongly Disagree*) to 7 (*Very Strongly Agree*). For items one and three, respondents could also select the options *I do not know* and *Not applicable*.

#### 2.4.3. Respondents’ research background

Two items asked respondents about their research background. The first item asked respondents to categorize their main research approach into either (1) hypothesis testing, (2) estimation, (3) modelling/simulations, (4) qualitative research or (5) other. The second item asked respondents to write down their specific research background (e.g. developmental psychology) as free text.

### 2.5. Procedure

Responses from the preregistration group were elicited by contacting all authors in our database (including the ones who participated in the pilot survey). Then, for each author in the preregistration group, we contacted up to five authors who published a non-preregistered empirical study in the same journal, volume, and issue. When we did not reach the desired sample size for the non-preregistration group, we proceeded to contact authors who had published in previous issues of the journals. This procedure was repeated several times and stopped when we had invited almost 2000 authors to our study. The decision to discontinue data collection deviates from our preregistered sampling plan but was motivated by the limitations of time and resources.

In the main survey, respondents were first asked to indicate if they had ever (1) preregistered a study that was not published; (2) preregistered a study that was published; (3) published a study that was neither preregistered nor a Registered Report; (4) created a Registered Report that was not published or (5) published a Registered Report. Based on their answers, the respondents were assigned to groups. Respondents were assigned to the preregistration group if they had published both preregistered and non-preregistered studies (i.e. they answered ‘yes’ to both option 2 and 3). Respondents were assigned to the non-preregistration group if they had published exclusively non-preregistered studies (i.e. answered ‘yes’ to option 3 and ‘no’ to all other options). In accordance with the preregistration plan, we only analyse and report data from these two groups.

Respondents then answered the remaining survey items and one intermediate attention check item (i.e. 2 + 2 = ?). The survey items and the attention check were presented in fixed order to the participants. The median amount of time respondents took to fill out the questionnaire was 3 min and 18 s.

### 2.6. Data exclusions

As preregistered, we excluded respondents if (1) they were assigned neither to the preregistered group nor to the non-preregistered group (*n* = 99); (2) they did not answer all questions in the survey (*n* = 23); (3) they failed the attention check (*n* = 18); (4) they indicated in the comment section that they could not provide adequate responses or they did not accept the informed consent form (*n* = 0).^2^ In total, we received 495 responses to our survey. After exclusion, 355 responses remained for the analysis. Of these, 299 responses came from the preregistration group and 56 responses came from the non-preregistration group.

### 2.7. Analysis

**Figure 1.**
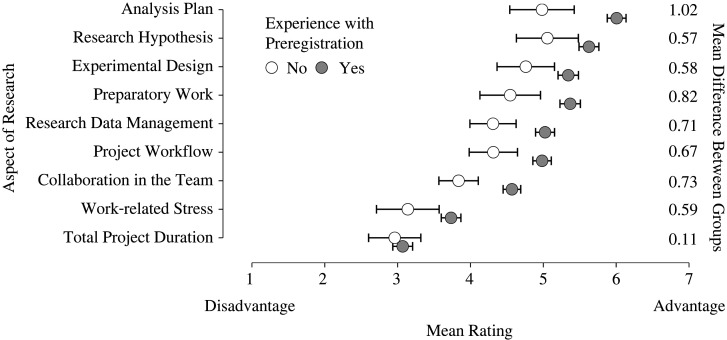
Respondents’ opinion on how preregistration influenced different aspects of the research process. Grey dots represent the mean ratings from respondents who have experience with preregistration and white dots represent the mean ratings from respondents who have no experience with preregistration. The square skewers represent 95% confidence intervals. Ratings above and below 4 indicate that preregistration helped and harmed a certain research aspect, respectively.

**Figure 2.**
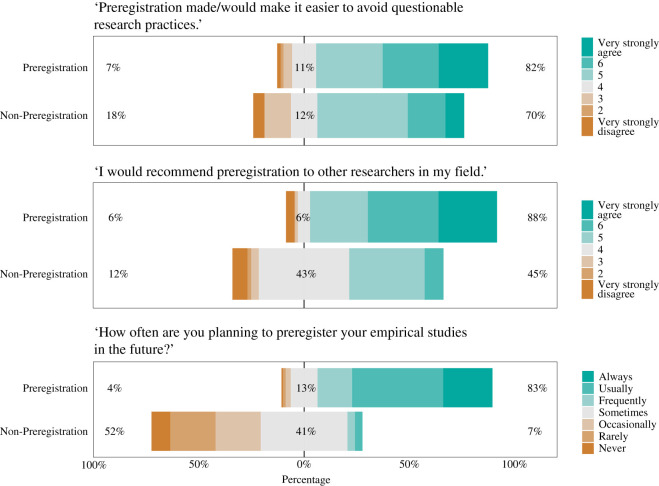
Respondents’ general opinion about preregistration. The top bar represents answers from respondents who have experience with preregistration, and the bottom bar represents answers from respondents who have no experience with preregistration. For each survey question, the number to the left of the data bar (in brown/orange) indicates the percentage who (slightly or strongly) disagreed or who would recommend preregistration occasionally or less frequently. The number in the centre of the data bar (in grey) indicates the percentage who responded with ‘neither agree or disagree’ or ‘neutral’. The number to the right of the data bar (in green/blue) indicates the percentage who (slightly or strongly) agreed or who would recommend preregistration frequently or more.

This is an exploratory study and therefore we present our results mainly through descriptive statistics. For the questions relating to nine aspects of the research process, we report both the means and 95% confidence intervals (figure 1). Note that the presence of confidence intervals deviates from our preregistration, which stated that no inferential procedure was going to be used.^3^ For the questions on the respondents’ opinion on preregistration, we visualize the frequency distributions of the survey responses (figure 2). We preregistered the intention to compare, both within the preregistration group and non-preregistration group, the answers of those who choose hypothesis testing as their empirical approach to the answers of those who choose a different approach (i.e. estimation, modelling/simulations, qualitative research, or other). Due to low response rate in the non-preregistration group, we could execute the intended comparison only within the preregistration group (as the sample size in the non-preregistration group was simply too small). We present the results of this comparison in appendix B. To foreshadow the results, the answers from the hypothesis testing group did not differ notably from those of the other group. For our analyses, we excluded responses that indicated *I do not know* and *Not applicable*. Finally, we compared the responses of the preregistration and non-registration group with respondents who reported having experience with preregistration but were not (yet) able to publish the studies they preregistered. This comparison was not preregistered but was suggested by the relatively high number of respondents that could not be assigned to either the preregistration or the non-registration group (*n* = 99). The results, reported in appendix C, show that the perceptions of researchers with unpublished preregistrations fall in between those with published preregistrations and the group without preregistration experience.

## 3. Results

### 3.1. Sample characteristics

We first sent 487 e-mail invitations to our contact database of researchers with experience in preregistration (see the Method section for a description). Out of these 487 e-mails, 30 bounced (i.e. there was an automatic failure to deliver the e-mail, for instance, because an address was no longer active), yielding a total of 457 successfully delivered requests. Removing incomplete surveys and respondents who failed the attention check left a total sample of 299 respondents who had experience with preregistration (i.e. a response rate of 65.43%).

Next, we invited a total of 1999 researchers who had published only non-preregistered studies. Out of these 1999 e-mails, 146 bounced, yielding a total of 1853 successfully delivered requests. The response rate for the non-preregistration group was lower than anticipated; receiving 56 responses from 1999 authors yields a response rate of only 2.80%. Due to this low response rate, we were unable to reach the preregistered target sample size, that is, for the non-preregistration group we only reached 18.7% of the number of responses from the preregistration group instead of the preregistered target of 90%.

Most respondents had a background in psychological science. Specifically, out of the 389 reported research backgrounds (some respondents reported more than one), 112 could be classified as social psychology (28.79%), 104 as experimental and cognitive psychology (26.74%), 36 as developmental and educational psychology (9.25%), 32 as personality psychology (8.23%), 17 as neurophysiology and physiological psychology (4.37%), 15 as applied psychology (3.86%), 12 as clinical psychology (3.08%) and 4 as methodology and statistics (1.03%). The remaining 57 responses (14.7%) could not be categorized into one of the areas above (e.g. anaesthesiology).

Out of the combined total of 355 respondents, 291 respondents indicated that hypothesis testing was their primary research approach, 21 indicated estimation, 25 indicated modelling/simulations, 3 indicated qualitative research and 15 respondents indicated other approaches.

#### 3.1.1. Nine aspects of research process

Figure 1 illustrates how preregistration was perceived to influence the nine different aspects of the research process. The specific breakdown of the answers to the individual questions is shown in table 3. Overall, both groups have a positive opinion on how preregistration influenced or would influence the different aspects of the research process, with the preregistration group generally being more positive than the non-preregistration group. Specifically, respondents were most positive about the benefits of preregistration regarding the analysis plan, the hypotheses, and the study design. For two aspects, however, respondents perceived preregistration to be disadvantageous: specifically, respondents indicated that preregistration would increase both work-related stress and total project duration.

**Table 3.** Per group, the mean ratings and 95% confidence intervals for each individual aspect on the research workflow measured on a 7-point rating scale, as well as the number of respondents answering *I do not know* or *Not applicable* on each aspect.

			no. respondents	
aspect	experience with preregistration	rating	*"I do not know"*	*"Not applicable"*
analysis plan	yes	*M* = 6.01 [5.88, 6.14]	0	0
	no	*M* = 4.98 [4.54, 5.42]	1	0
research hypothesis	yes	*M* = 5.63 [5.49, 5.77]	1	1
	no	*M* = 5.06 [4.63, 5.49]	2	0
experimental design	yes	*M* = 5.34 [5.20, 5.48]	1	3
	no	*M* = 4.76 [4.37, 5.15]	1	1
preparatory work	yes	*M* = 5.37 [5.23, 5.51]	2	4
	no	*M* = 4.55 [4.14, 4.96]	1	0
research data management	yes	*M* = 5.02 [4.89, 5.15]	2	4
	no	*M* = 4.31 [3.99, 4.63]	1	0
project workflow	yes	*M* = 4.98 [4.85, 5.11]	5	2
	no	*M* = 4.31 [3.98, 4.64]	5	0
collaboration in the team	yes	*M* = 4.57 [4.45, 4.69]	5	4
	no	*M* = 3.84 [3.57, 4.11]	6	1
work-related stress	yes	*M* = 3.73 [3.59, 3.87]	5	1
	no	*M* = 3.14 [2.71, 3.57]	6	0
total project duration	yes	*M* = 3.07 [2.93, 3.21]	11	1
	no	*M* = 2.96 [2.60, 3.32]	6	0

*Note.* Square brackets indicate the 95% confidence interval for the ratings. *N* = 299 for preregistration group, *N* = 56 for non-preregistration group.

The preregistration group and the non-preregistration group differed mostly in their opinion on how preregistration influences the analysis plan and preparatory work. Although both groups reported that preregistration would benefit these aspects, respondents with preregistration experience were more enthusiastic. That is, the preregistration group reported that preregistration had made the analysis plan more thought-through (*M* = 6.01 [5.88, 6.14] versus *M* = 4.98 [4.54, 5.42]) and that preregistration improved the preparatory work of the project (*M* = 5.37 [5.23, 5.51] versus *M* = 4.55 [4.14, 4.96]).

In four aspects of the research process, that is, research hypothesis, experimental design, work-related stress and total project duration, the groups showed the smallest differences of opinion. Whereas both groups perceived preregistration to benefit the experimental design (*M* = 5.34 [5.20, 5.48] in the preregistration group versus *M* = 4.76 [4.37, 5.15] in the non-preregistration group) and the research hypothesis (*M* = 5.63 [5.49, 5.77] in the preregistration group versus *M* = 5.06 [4.63, 5.49] in the non-preregistration group), preregistration was perceived to be a disadvantage with respect to work-related stress (*M* = 3.73 [3.59, 3.87] in the preregistration group versus *M* = 3.14 [2.71, 3.57] in the non-preregistration group) and total project duration (*M* = 3.07 [2.93, 3.21] in the preregistration group versus *M* = 2.96 [2.60, 3.32] in the non-preregistration group).

One aspect in which both groups gave qualitative different answers based on the group means was the influence of preregistration on the collaboration in the team. While respondents in the preregistration group indicated that it had improved the collaboration in the team (*M* = 4.57 [4.45, 4.69]), respondents in the non-preregistration group indicated that it would be a slight disadvantage (*M* = 3.84 [3.57, 4.11]).

#### 3.1.2. Opinion about preregistration

Figure 2 summarizes the general opinion about preregistration among respondents. The vast majority of respondents in the preregistration group had a positive overall opinion about the practice. Eighty-two per cent of respondents agreed with the statement that compared to their non-preregistered work, preregistration had helped avoid questionable research practices. For this statement, no researcher responded with *Not applicable* and one researcher responded with *I do not know*. A quarter of respondents (23.5%; 70 of 298) reported to *very strongly agree* with this statement, which may suggest that other researchers have at least some reservations that preregistration is the ultimate solution to preventing questionable research practices.

In addition, 88% of respondents would recommend the practice to other researchers in their field. No researchers indicated *I do not know* or *Not applicable* to this statement. Finally, 83% of the respondents in the preregistration group would consider preregistration in their future work. The results are somewhat more ambiguous in the group of respondents without preregistration experience. Although 70% agreed with the statement that preregistration would make it easier to avoid questionable research practices (with only 9%, that is, 5 of 56, indicating to *very strongly agree* with the statement), only 45% would recommend the practice to other researchers in their field. No researchers in the non-preregistration group indicated *I do not know* or *Not applicable* to these statements. Preregistration is also not seen as desirable for future research projects: only 7% in the non-preregistration group would consider this practice in their future work.

## 4. Constraints on generality

The present study surveyed researchers who have experience with preregistering studies and those who did not. Our sample consisted exclusively of researchers in the field of psychology, presumably from differing career stages. The biggest concern regarding generalizability is that our sample was subject to self-selection. Since participation in the survey was voluntary, researchers who already had a strong opinion about preregistration might have been more likely than others to participate.

Since the proportion of respondents in the preregistration group was relatively high with 65.43%, we assume that our sample therefore reflects the population of these researchers relatively well. Therefore, we expect the results from respondents in the preregistration group to generalize to other researchers within the field of psychology who have experience with preregistration.

The results from the non-preregistration group, on the other hand, might generalize poorly to other researchers in the field since the proportion of respondents in the non-preregistration group was very low (2.80%). In the field of meta-science, low response rates are no exception: Field *et al.* [20], for instance, achieved a response rate of 6%, Malički *et al.* [7] a response rate of 4.9%. Gopalakrishna *et al.* [9], on the other hand, achieved an exceptional high response rate of over 21%. The low response rate in our study suggests that for the non-preregistration group self-selection might have had a stronger effect on the results. That is, it may be that predominantly researchers with strong opinions about preregistration responded to this survey, rather than those who felt neutral about the practice. However, it should be noted that despite the low response rates in the non-preregistration group the general response pattern (that is, the ranking of the research aspects) is consistent in both groups. This systematicity might indicate that we were not dealing with a select subgroup, or at least that the opinions of the select subgroup do not differ much from researchers with preregistration experience.

## 5. Discussion

In the last decade, preregistration has been advocated as a tool to prevent researchers’ biases and expectations from contaminating the statistical analyses. It has also been argued that preregistration may have secondary effects on the research process. The current study sought to unveil these expectations and experiences.

Our results suggest that researchers find preregistration to benefit their work in most aspects of the research process. Researchers in our sample reported that preregistration improved the theoretical aspects of the project (e.g. the generation of the research hypothesis, the research design, and the analysis plan) as well as practical aspects of the project (e.g. the design and execution of pilot or simulation studies, and the general project workflow). However, disadvantages of preregistration also became apparent; researchers reported that preregistering a study had increased or was expected to increase the total project duration and the work-related stress.

The increase in time and effort to publish a preregistered study had been acknowledged in the literature (e.g. [8,18]). However, some statements made previously on the influence of preregistration on work-related stress contradict our findings. For instance, Frankenhuis & Nettle ([27], p. 441) write: ‘From hearsay and our own experience, we think that scholars find it relaxing not to have to make [·· ·] critical decisions after having seen the data, accompanied by a lingering sense of guilt, while cognizant of some of their biases and frustratingly unaware of others.’

Although researchers with preregistration experience reported that this practice increased the total project duration and work-related stress, the vast majority of this group also indicated that they would recommend the practice to other researchers in their field and continue to use it for their own research projects. As one respondent mentioned in the free-text comments: ‘Pre-Reg improves quality, which causes more work, as it should be’. For researchers without preregistration experience, the equation does not seem to add up: the majority of this group would not recommend the practice to their peers or consider this practice for themselves in the future.

We identified three limitations of the study. The first limitation is that our survey was based on self-report and therefore cannot demonstrate the extent to which the perceived secondary effects of preregistration correspond to its actual secondary effects. To answer this question, workflows and manuscripts from preregistered and non-preregistered studies would need to be evaluated by independent researchers. To avoid potential sample bias, this could be done in an experimental setting: research teams could be randomly assigned to the preregistration group or the non-preregistration group and be instructed to design and conduct a study to answer the same research question. An appropriate setting for such an experiment would be, for instance, a multi-laboratory project conducting conceptual replications.

The second limitation concerns the low response rate and small sample size of the non-preregistration group. One explanation for this could be that, of the researchers who do not have experience with preregistration, only those who already have strong opinions about the practice are inclined to answer a preregistration survey. For researchers who are neutral about preregistration, a survey on this topic may simply not be interesting enough. Perhaps the researchers were also averse to the way we approached them, perhaps our invitation email was worded too strongly in favour of preregistration (our invitation letters can be accessed at https://osf.io/t376k/), or it was off-putting that the survey was signed by known proponents of preregistration (i.e. the email was signed by all co-authors and sent from B. A.’s private email account). In fact, the meta-scientific survey study by Gopalakrishna *et al.* [9], which had a remarkably high response rate of 21.1%, had the data collection conducted by an international market research company.

The last limitation concerns the wording of the items in this survey. In the current study, respondents in the preregistration group were asked about their experiences with their previous research projects, whereas respondents in the non-preregistration group were asked about their expectations for future research. We opted for this phrasing as we intended to capture the actual effects of preregistration on workflow in the preregistration group, which might arguably be less subject to bias than expected secondary effects. However, this wording may have reduced comparability between the two groups. Future research might therefore consider asking respondents in the preregistration group additionally about their expectations for future projects.

How can researchers benefit from the secondary effects of preregistration? Whether or not preregistration improves the secondary aspects of the research process depends largely on the quality of the preregistration document. That is, the thoroughness of the preregistration protocol determines how carefully researchers need to think about the study design and analysis plan. A high-quality preregistration document features detailed information about the experimental conditions, the materials and stimuli used, and a comprehensive analysis plan (preferably featuring a mock dataset and analysis code). To ensure that preregistration protocols meet these quality standards without considerable extra effort, researchers can fall back on a range of checklists, guidelines, and preregistration templates. Preregistration templates for the standard experimental framework can be found, for instance, on the websites aspredicted.org or on the Open Science Framework (https://osf.io/zab38/). The number of preregistration templates and tutorials for other research areas and more complex methods is increasing and includes cognitive modelling [28], secondary data analysis of pre-existing data [29,30], studies using experience sampling methods [31], and qualitative research [32,33]. Finally, the recently developed Transparency Checklist is a quick way to check whether the preregistration and the accompanying paper comply with the current transparency standards [34].

Some researchers might also prefer alternative methods to preregistration. One of these alternatives that allows for more flexibility while still safeguarding the confirmatory status of the research is analysis blinding [35–38]. With analysis blinding, researchers are in principle not required to write a preregistration document. Instead, they collect their experiment data as usual and develop their analysis plan based on an altered version of the data in which the effect of interest is hidden (e.g. by shuffling the outcome variable). Another alternative would be to minimize bias by trying to map out the uncertainty in the analyses with various statistical practices [39]. For instance, researchers could explore the entire universe of outcomes through multiverse analyses (in which all theoretically sensible data-preprocessing steps are explored; [40]) or multi-analysts approaches (in which multiple analysis teams answer the same research question based on the same dataset; e.g. [41,42]).

Our survey shows that researchers see preregistration as beneficial to their research workflow and the overall quality of their work. We consider this to be a welcome byproduct of the practice: one ensures the confirmatory status of one’s analyses and experiences an improvement in practical aspects of one’s workflow. However, this does not mean preregistration is the preferred means of improving workflow; other methods are probably better suited for this purpose. For instance, the recently proposed theory construction methodology by Borsboom *et al.* [43] was developed to assist researchers in identifying and linking empirical phenomena, in constructing and mathematically representing theories, and evaluating these theories. As such, this methodology could likewise improve the quality of the analysis plan, research hypothesis, preparatory work, and experimental design, presumably to a greater extent than preregistration can. Similarly, we expect that the Registered Report format, which entails close scrutiny and revision of theory, experimental design, and analysis plan by independent scholars, could achieve greater secondary benefits than preregistration alone.

Researchers who have strong reservations about preregistration, whether conceptual or practical, are unlikely to be persuaded by the experiences of their peers. However, those who are still undecided whether the practice is worth trying may be convinced by its practical advantages. To them we say: try preregistration and form your own opinion about its possible advantages and disadvantages.

In order for preregistration to truly become the norm in psychology, it is necessary for journals, institutions, and funding agencies to provide sufficient incentives for researchers. In addition, we believe that the research culture still needs to evolve: in terms of ensuring preregistration is considered good research practice in individual labs, but also in terms of making sure that studies that cannot be preregistered are not stigmatized. Some of the negative experiences that have been made with preregistration could possibly be reduced with methodological advancements. For instance, combining preregistration with analysis blinding might increase the adherence to analysis plans. Better-structured templates could improve the efficiency of the method, and more precise instructions could increase the accuracy of preregistration, thereby also increasing its effectiveness.

### 5.1. Concluding remarks

The aim of this study was to obtain an overview of the experienced and expected advantages and disadvantages of the practice of preregistration. Our survey shows that relying on intuition alone when developing open research practices might not be enough. Only if we know how the conceptual advantage of preregistration weighs against the individual experienced benefits and challenges can we find suitable means to improve the methodology so that it finds wider acceptance among researchers.

## Figures and Tables

**Figure 3.**
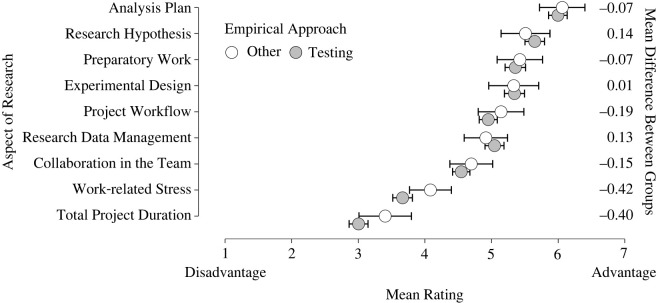
Respondents’ opinion on how preregistration influenced different aspects of the research process. Grey dots represent the mean ratings from the respondents who indicated that their empirical approach was hypothesis testing and white dots represent the mean ratings from respondents who indicated a different empirical approach. The square skewers represent 95% confidence intervals. Ratings above and below 4 indicate that preregistration helped or harmed a certain research aspect, respectively.

**Figure 4.**
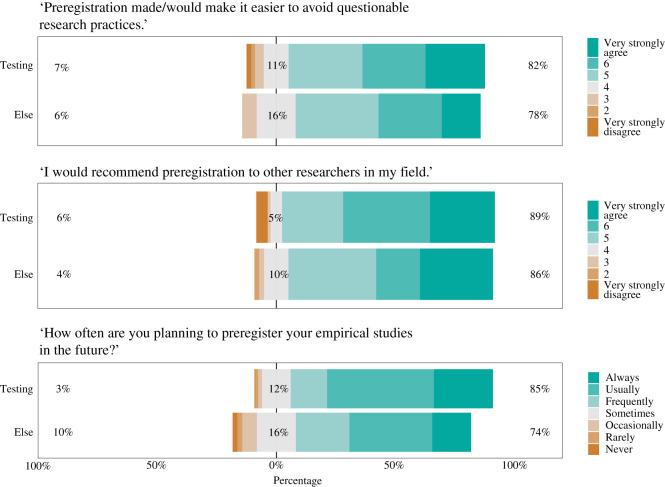
Respondents’ general opinion about preregistration. The top bar represents answers from respondents whose main empirical approach was hypothesis-testing, the bottom bar represents answers from respondents whose main empirical approach was different. For each survey question, the number to the left of the data bar (in brown/orange) indicates the percentage who (slightly or strongly) disagreed or who would recommend preregistration occasionally or less frequently. The number in the centre of the data bar (in grey) indicates the percentage who responded with ‘neither agree or disagree’ or ‘neutral’. The number to the right of the data bar (in green/blue) indicates the percentage who (slightly or strongly) agreed or who would recommend preregistration frequently or more.

**Figure 5.**
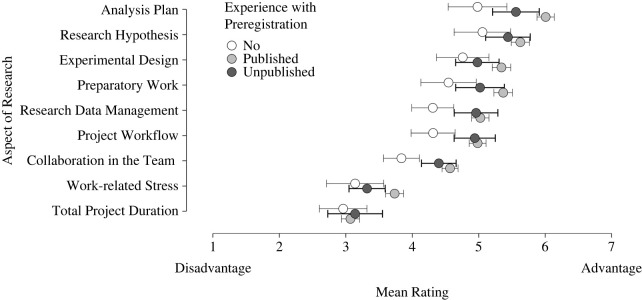
Respondents’ opinion on how preregistration influenced different aspects of the research process. Dark grey dots represent the mean ratings from the *n* = 55 respondents who have experience solely with unpublished preregistrations. White dots represent the mean ratings from the *n* = 56 respondents who have no experience with preregistration, light grey dots represent the mean ratings from *n* = 299 respondents who have published a preregistration. The square skewers represent 95% confidence intervals. Ratings above and below 4 indicate that preregistration helped or harmed a certain research aspect, respectively.

**Figure 6.**
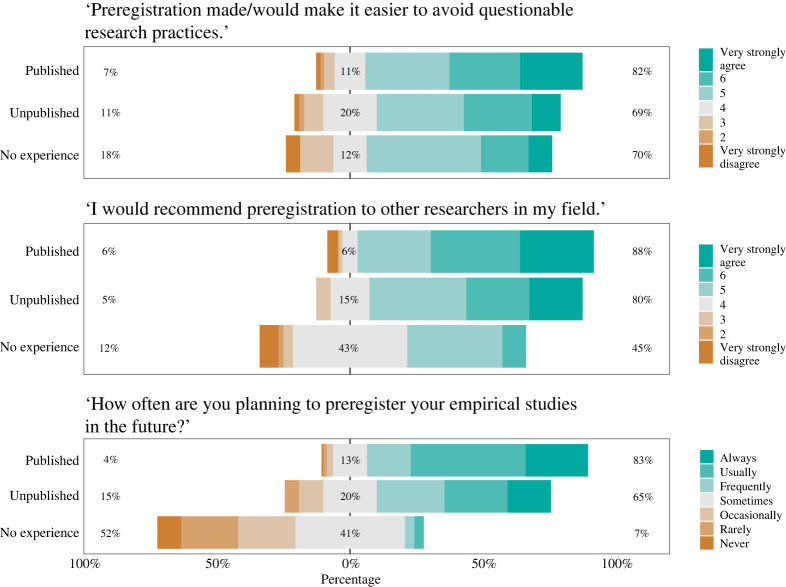
Respondents’ general opinion about preregistration. The top bar represents answers from respondents who have published a preregistration, the middle bar represents answers from respondents who have experience with unpublished preregistrations, and the bottom bar represents answers from respondents who have no experience with preregistration. For each survey question, the number to the left of the data bar (in brown/orange) indicates the percentage who (slightly or strongly) disagreed or who would recommend preregistration occasionally or less frequently. The number in the centre of the data bar (in grey) indicates the percentage who responded with ‘neither agree or disagree’ or ‘neutral’. The number to the right of the data bar (in green/blue) indicates the percentage who (slightly or strongly) agreed or who would recommend preregistration frequently or more.

## Data Availability

The current study was preregistered on the Open Science Framework; in our project folder (https://osf.io/jcdvb/), readers can access the preregistration, as well as all materials for both the pilot and the main survey, the contact database used for the main survey, the anonymized raw and processed data (including relevant documentation), and the R code to conduct all analyses (including all figures). In our datasets, identifying information such as names and affiliations of the respondents were removed. Any deviations from the preregistration are mentioned in this manuscript. Note that we removed email addresses from the contact database for privacy reasons.

## Footnotes

1 The catalogue also included, for instance, the statement that authors should report the availability of all data, materials and codes (83% agreement across all fields) and the statement that journals should encourage the submission of replication studies (61% agreement across all fields).

2 Note that exclusion criterion (1) also pertains to respondents who indicated that their experience with preregistration related solely to Registered Reports (i.e. they responded ‘yes’ to options 4 or 5, but ‘no’ to all other options). We decided to exclude these respondents (*n* = 2) since we suspected that secondary benefits of the Registered Reports format might be influenced in large part by the extensive review process.

3 Since we had not made any predictions about our data, we did not preregister inferential procedures, but found it informative to display the statistical uncertainty associated with the mean ratings.

## Appendix A. Summary of free-text comments

In our survey, respondents both completed the questionnaire and had the opportunity to provide comments on preregistration in an open-ended format. This section summarizes these comments. For this purpose, the authors A.S. and M.K. have divided the comments into different topics and evaluated whether they were positive, negative, or neutral statements. Comments on other topics than preregistration (e.g. comments on the survey) are not here. The full list of comments is available in our online repository at https://osf.io/5ytpk/. We would like to emphasize that the results should be interpreted with caution. The comments evaluated below are based on only a fraction of the respondents. Therefore, the overview given here is not necessarily representative of the opinions in our sample.

Seventy-eight researchers provided us with free-text comments on preregistration. These comments highlighted both the advantages and disadvantages of preregistration: 20 comments were exclusively positive, 22 comments were negative and 36 comments were mixed. The comments could be categorized roughly into five topics. The topics were (1) the additional workload of preregistration (mentioned by *n* = 24 respondents); (2) the effectiveness of preregistration in solving the crisis of confidence (mentioned by *n* = 19); (3) the impact of preregistration on one’s career (mentioned by *n* = 16 respondents); (4) how preregistration might contribute to inequality and stigmatization in different research areas (mentioned by *n* = 13 respondents); (5) and the difficulties in the compliance with the preregistration protocol (mentioned by *n* = 11 respondents).

**A.1. Additional workload of preregistration: harder, but worthwhile?**

Proponents of preregistration argue that despite the additional workload preregistration cases, it is still ‘worthwhile’ (e.g. [8]). But do researchers agree with that statement? Not necessarily. From the *n* = 24 respondents who mentioned the additional workload, *n* = 11 respondents believed that preregistration was harder and worthwhile while seven respondents believed that it was harder, but not worthwhile—six respondents mentioned the increased workload without any further judgement. For respondents who thought preregistration was hard, but worthwhile, the added benefit of improved overall quality outweighed the added workload or was perceived as a necessary consequence (e.g. Pre-Reg improves quality, which causes more work, as it should be). Others recognized the theoretical value of preregistration, but did not see the benefits translating into practice. For instance, one respondent wrote: ‘I think preregistration is great in theory, but in practice it serves only to increase the red tape and time until publication. In today’s hyper-competitive publish-or-perish job market, it amounts to time wasted’. The added time it takes to write a preregistration even seems to scare researchers from trying out the practice: ‘I understand the importance of [preregistration], but the amount of time and effort needed to preregister is probably the biggest reason I have avoided it in the past’.

**A.2. Effectiveness of preregistration in solving the crisis of confidence**

Nineteen respondents mentioned that preregistration improved the credibility of their results and the overall quality of their work. Seven respondents, however, questioned whether preregistration was a suitable tool to address the crisis of confidence. Besides the need for theory development and exploratory research, lack of methodological knowledge, and possibilities to cheat the system (by creating multiple preregistration documents) were mentioned. In addition, multiple respondents criticized the incentive structure in science, which is designed to reward research output and thus discourages the adoption of preregistration (e.g. [U]nless we rid science from the publication for-profit industry and educate our universities not to use the incentive structure that still very much determines who gets hired and who gets promoted based on where researchers publish rather than what they publish, I am afraid we have left the big elephant in the room untouched.; [T]he speed at which our institutions expect us to pump through graduate students often means that pre-reg cannot happen for their work [·· ].).

**A.3. Influence of preregistration on the career**

Sixteen respondents reported how preregistration influenced their career. Two respondents indicated that embracing open science practices helped their career, for instance, by giving them an advantage during the hiring process. With respect to research output, five respondents reported that publishing preregistered studies was easier while six respondents reported that it was harder. The main arguments as to why preregistered articles were easier to publish was that the respondents felt that a preregistration was expected by the journals, or they described that the ‘in principle acceptance’ granted for Registered Reports made the publication process easier. On the other hand, respondents also described how reviewers or editors rejected papers if authors did not adhere to their preregistered plan, or that they pushed them towards rewriting their manuscripts to present polished narratives (e.g. [R]eviewers sometimes have even criticized that I report non-significant results; [I] often encounter editors who still seem to want my team to change *a priori* aspects of manuscripts to better fit with a *we knew it all along* or in the context of competing hypothesis situations, favour the hypothesis that was ultimately supported by the data).

**A.4. Inequality and stigmatization**

In our survey, 13 respondents addressed the disadvantages preregistration can have in research fields outside of psychology and for descriptive and exploratory study designs. As mentioned by some respondents, when working in fields outside of psychology (e.g. animal research) or when the research area has interfaces with industry, preregistration is relatively unknown which makes preregistered studies harder to publish (e.g. ‘[· · ·] My field (animal research) is substantially behind the curve. To date, of the preregistered studies I have attempted to publish, no reviewer has commented on the preregistration as a positive aspect of the study [· · ·]. Rather, the reviewers who have mentioned it have used the preregistration to point out deviations (which we take care to explicitly point out in the methods) and thus has led to more challenges with publication rather than fewer. I am of the opinion that if I had submitted identical studies without preregistration, they would have been easier to publish. [· · ·]’)

In addition, respondents perceived that preregistration went to the detriment of descriptive and exploratory research. For instance, one respondent argues that confirmatory and preregistered experimental studies are currently perceived as ‘the gold standard [· · ·] which leaves behind other kinds of exploratory and descriptive studies.’ Another respondent argues that psychology ‘needs a clearer distinction between confirmatory and exploratory work, and wider recognition of the value of exploratory, descriptive research that can form the basis for well-specified hypotheses’. Lastly, five respondents critiqued that preregistration causes stigmatization for studies that have not been preregistered. In their comments, respondents critiqued that the reviewers often prematurely condemn a non-preregistered study, without considering its individual peculiarities. As suggested by one of the respondents, the scientific community should place more emphasis on positive reinforcement rather than harsh judgement (e.g. I am still in favour of pre-registration and open science and I plan to pre-register the studies that I lead. At the same time, I wish that the movement was more moderate and based more on positive reinforcement).

**A.5. Problems with data exploration and compliance with the preregistration protocol**

Eleven respondents commented that preregistration would limit creativity, that it discourages researchers to explore the data and that adherence with the preregistration protocol was problematic, especially for early career researchers ‘who are still learning as they go’, or when working with complex models (e.g. ‘In my work it is hard or sometimes impossible to know how the data should be analysed before seeing its structure, distribution, etc.—and there is no way of accounting for every possibility in the prereg.’).

## Appendix B. Hypothesis testing and exploratory research

The following section takes a closer look at the responses within the preregistration group. Specifically, we were interested in whether a researcher’s empirical approach influences perceptions of preregistration, for instance, in that researchers who primarily test hypotheses (i.e. focusing mainly on the existence of an effect) view preregistration as more beneficial than researchers with other empirical approaches. Such alternative approaches include parameter estimation (focusing mainly on the size of an effect), qualitative research (focusing mainly on understanding an effect) or modelling/simulations (focusing mainly on development of statistical methods).

Within the preregistration group, 250 respondents indicated that hypothesis testing was their main empirical approach while 49 respondents indicated that their main empirical approach was a different one (e.g. estimation, modelling/simulations, qualitative research, other).

Figure 3 illustrates how preregistration was perceived to influence the nine different aspects of the research process. Overall, both groups have a positive opinion on how preregistration influenced the different aspects of the research process. The pattern resembles that of the preregistration group in general, with the analysis plan benefiting the most from preregistration while the total project duration and work-related stress have been negatively affected by the practice. Respondents who do hypothesis-testing seemed to be somewhat more negative than respondents with a different empirical approach. The biggest difference in opinion was regarding work-related stress. Here, the hypothesis-testing group perceived preregistration to be a disadvantage (*M* = 3.67 [3.52, 3.81]), while respondents with a different empirical approach were neutral (*M* = 4.08 [3.77, 4.40]).

Figure 4 illustrates the general opinion about preregistration among the respondents. The two groups do not show meaningful differences in opinion. In both groups, more than 75% agreed with the statement that compared to their non-preregistered work preregistration helped them avoid questionable research practices and more than 85% would recommend the practice to other researchers in their field. Finally, over 85% of the respondents who do hypothesis-testing would consider preregistration in their future work and 73% of the respondents with a different empirical approach would consider it in their future work.

## Appendix C. Published versus unpublished preregistrations

In our main results, all respondents in the preregistration group had at least one positive experience with preregistration in that they successfully published at least one preregistered article. In this section, we explore the attitudes of researchers who have not (yet) been able to publish the studies they preregistered. Specifically, we were interested to explore if this group experienced preregistration as particularly frustrating or whether they perceived the practice as positively as researchers who have successfully published a preregistration. This comparison was not preregistered.

From the 99 respondents who were assigned neither to the preregistration group nor to the non-preregistration group, 63 reported having experience with preregistration but have not published one (yet). Excluding the respondents who have experience with Registered Reports, this left a sample of 55 respondents (henceforth denoted as unpublished-preregistration group). Note that from these data, it is not possible to deduce why the researchers could not publish their preregistered studies. Their experiences could be based on ongoing studies, or perhaps on studies that were difficult to publish.

Figure 5 shows how respondents rated the effects of preregistration on the nine different aspects of the research process. Table 4 shows a more detailed overview of their responses. As in our previous results, respondents in the unpublished-preregistration group (dark grey dots) have a positive opinion on how preregistration influences the different aspects of the research process. The response pattern in this group resembles that of our main sample, depicted with white dots and light grey dots. The figure suggests that the opinions of respondents in the unpublished-preregistration group lie between those who have published preregistrations and those who have no preregistration experience. Concerning the aspects ‘research data management’, ‘project workflow’ and ‘collaboration in the team’, the group seems closer to the opinions of the preregistration group. In the aspect ‘work-related stress’, however, the group has a more negative attitude, similar to the non-preregistration group.

**Table 4.** For the 55 respondents in the unpublished-preregistration group, the table shows the mean ratings and 95% confidence intervals for each individual aspect on the research workflow measured on a 7-point rating scale, as well as the number of respondents answering *I do not know* or *Not applicable* on each aspect.

		no. respondents	
aspect	rating	*"I do not know"*	*"Not applicable"*
analysis plan	*M* = 5.56 [5.21, 5.91]	0	0
research hypothesis	*M* = 5.44 [5.10, 5.78]	0	0
preparatory work	*M* = 5.02 [4.65, 5.39]	1	0
experimental design	*M* = 4.98 [4.65, 5.31]	0	3
research data management	*M* = 4.96 [4.63, 5.29]	0	1
project workflow	*M* = 4.94 [4.63, 5.25]	0	1
collaboration in the team	*M* = 4.40 [4.14, 4.66]	1	2
work-related stress	*M* = 3.32 [3.05, 3.59]	2	0
total project duration	*M* = 3.14 [2.73, 3.55]	4	0

*Note.* Square brackets indicate the 95% confidence interval for the ratings.

Figure 6 illustrates the general opinion about preregistration among the respondents. Again, the opinions of respondents who have only unpublished preregistration experience lie between those who have published preregistrations and those who have no preregistration experience. More than 69% agreed with the statement that preregistration would help them avoid questionable research practices and 80% would recommend the practice to other researchers in their field. Unlike respondents in the non-preregistration group, the majority of respondents in the unpublished-preregistration group plans to use preregistration in future projects (7% versus 65%, respectively).

Overall, respondents in the unpublished-preregistration group do not seem to feel frustrated by the process of preregistration. At the same time, this group is somewhat less enthusiastic about the practice than the respondents who have already published a preregistered study.
